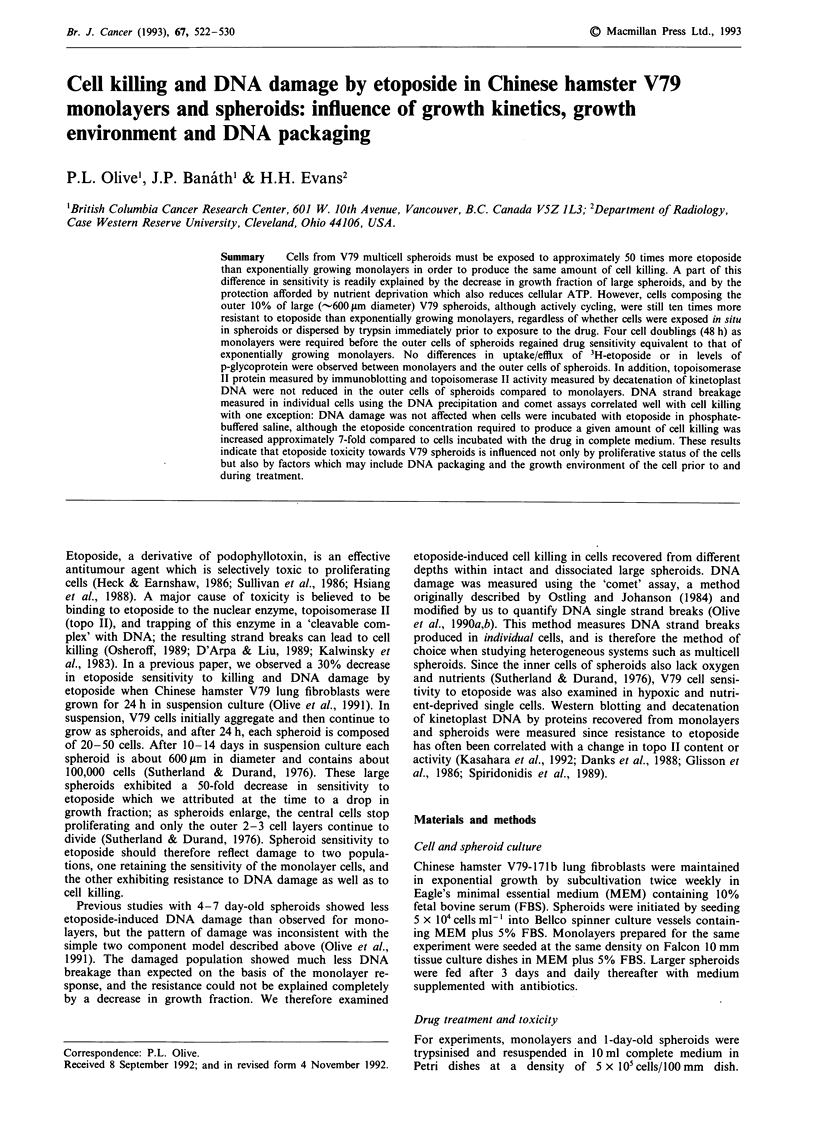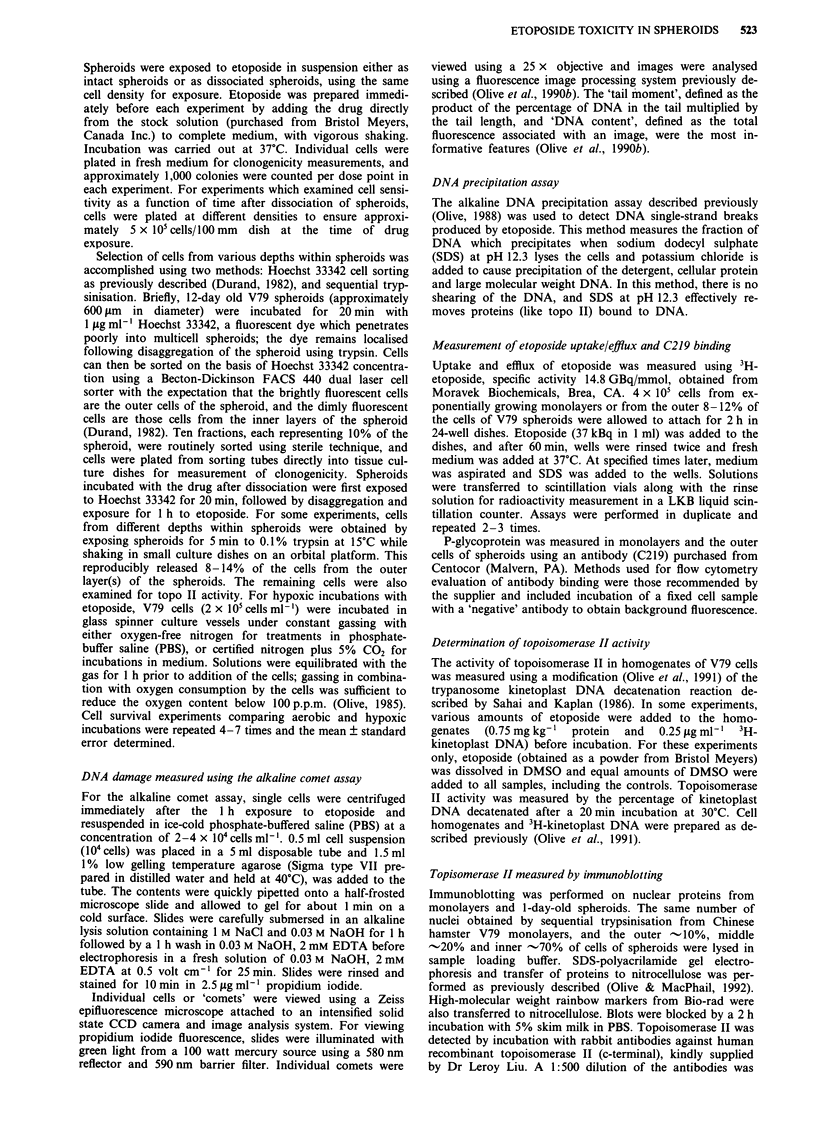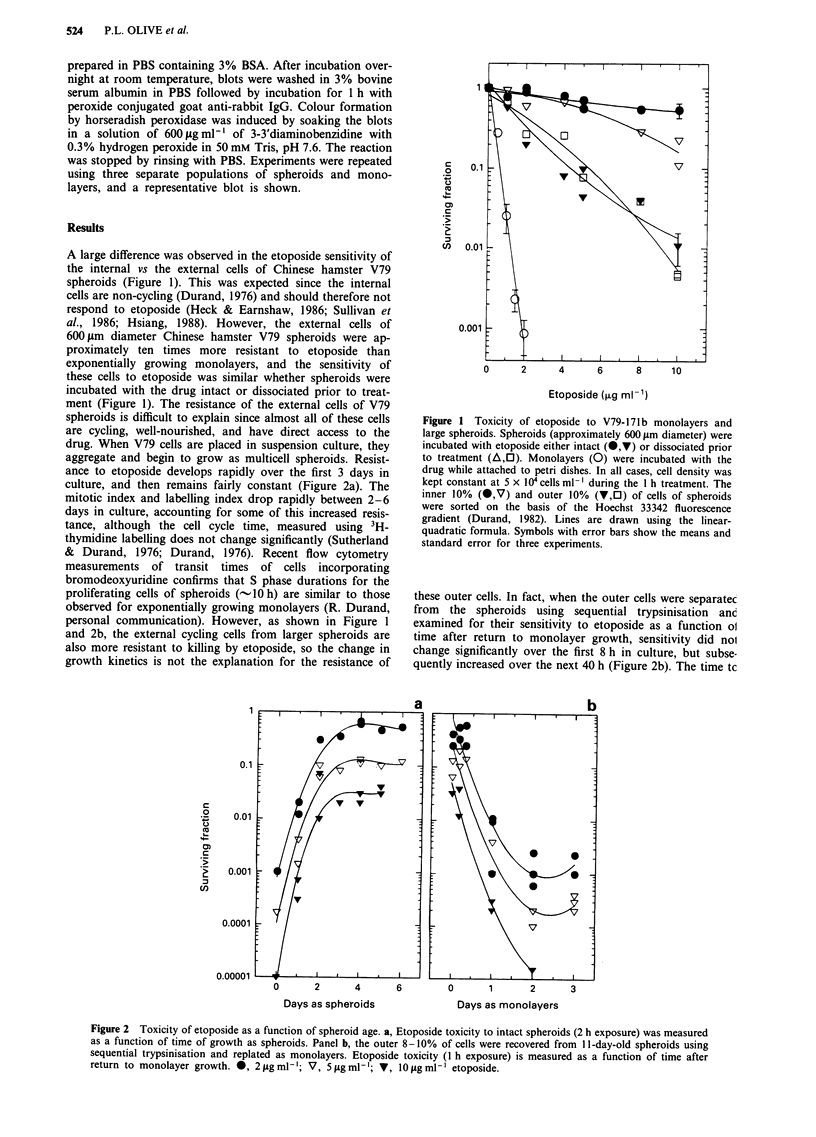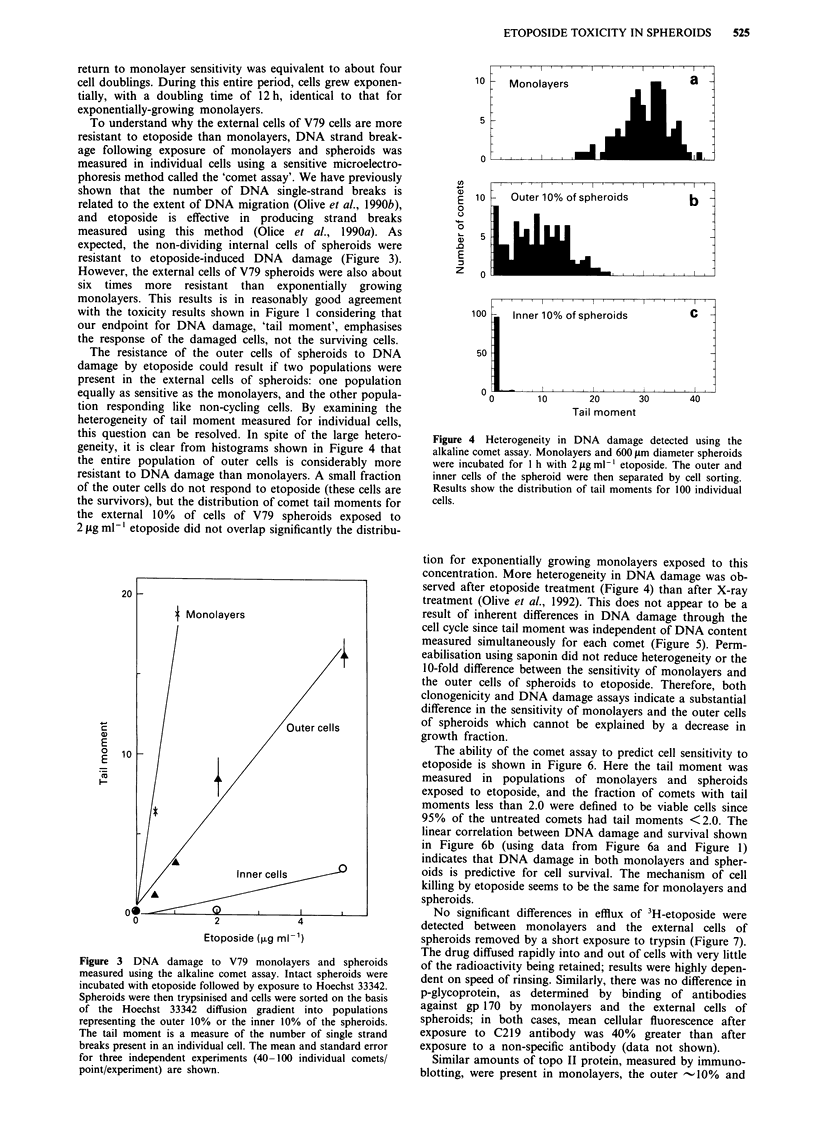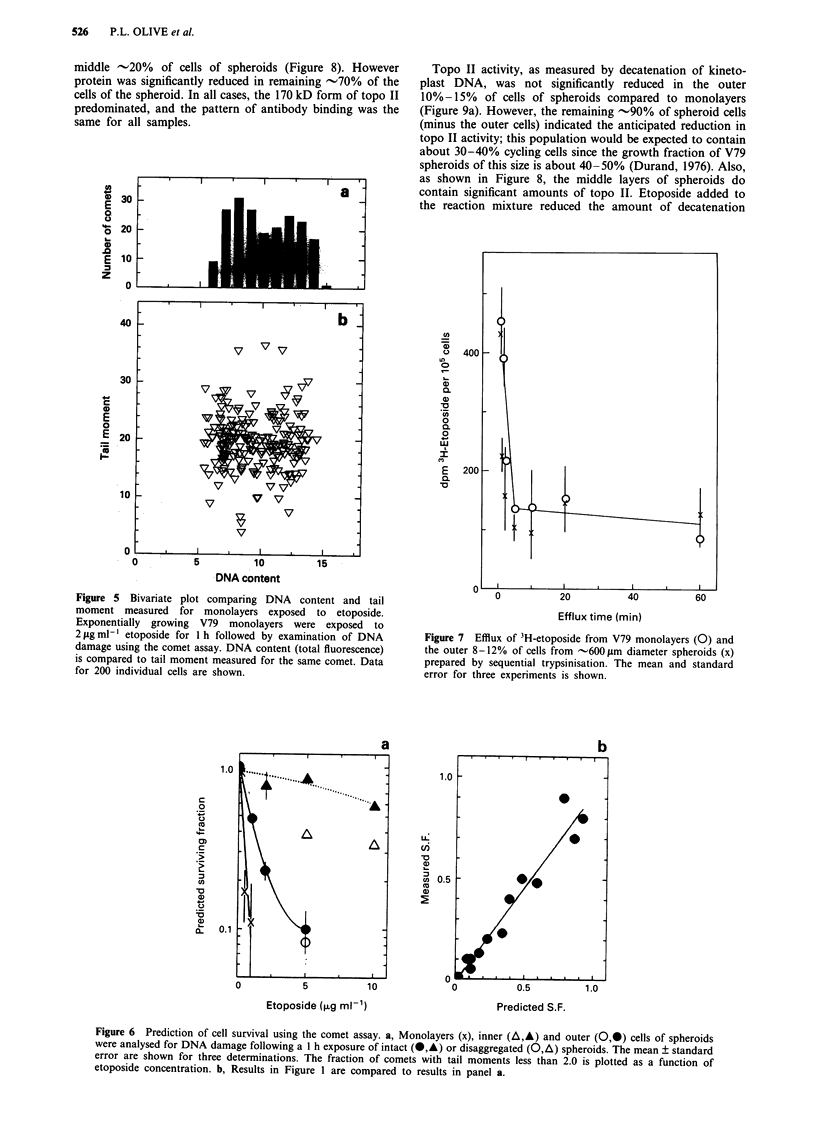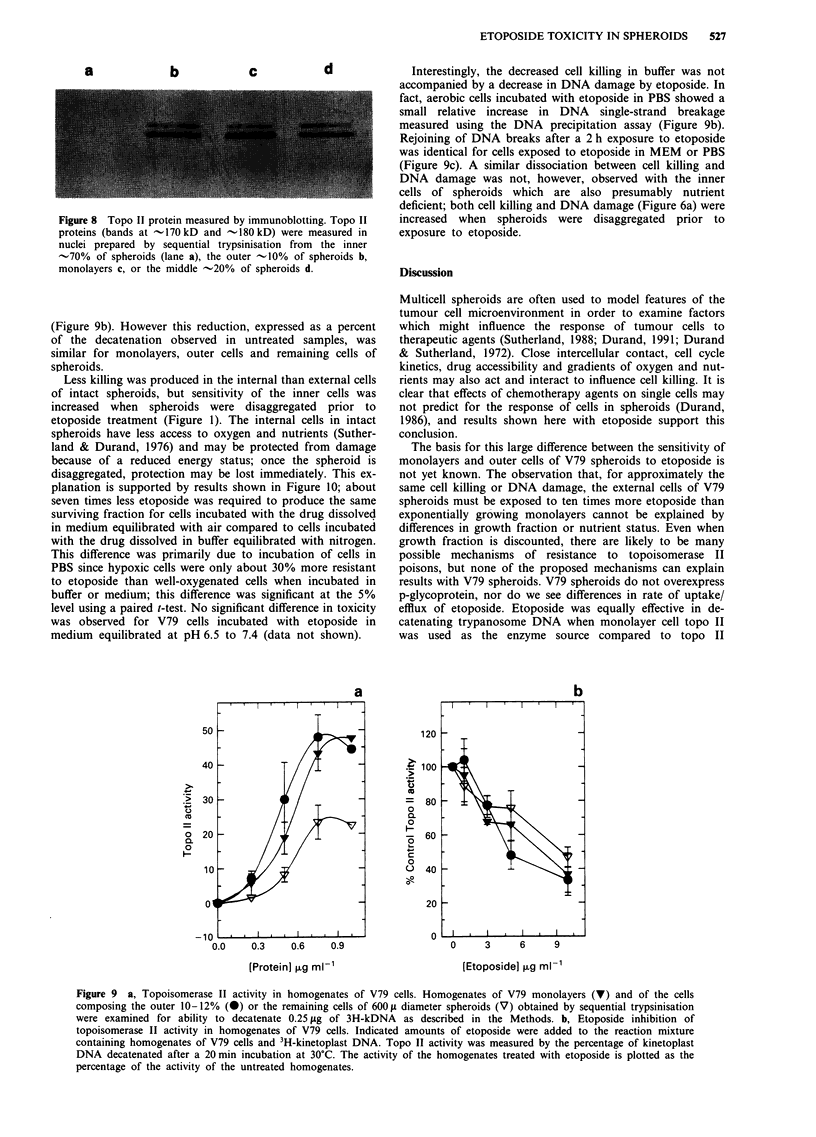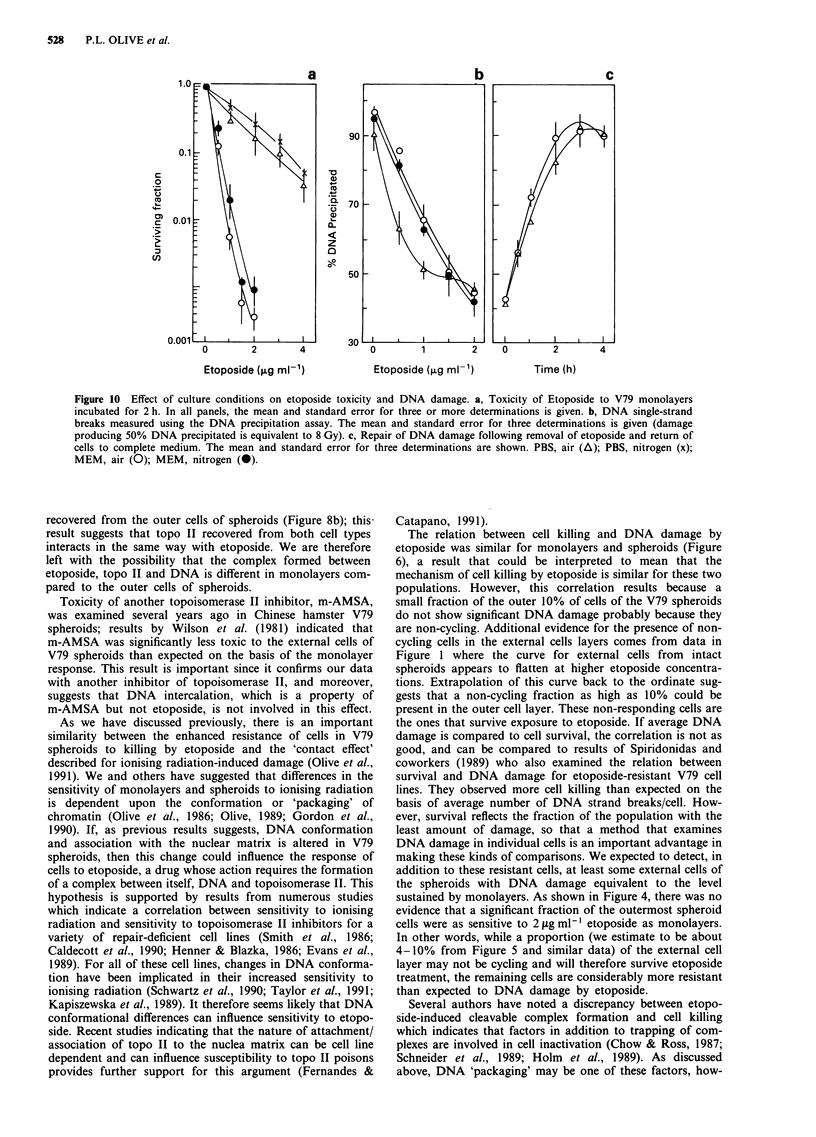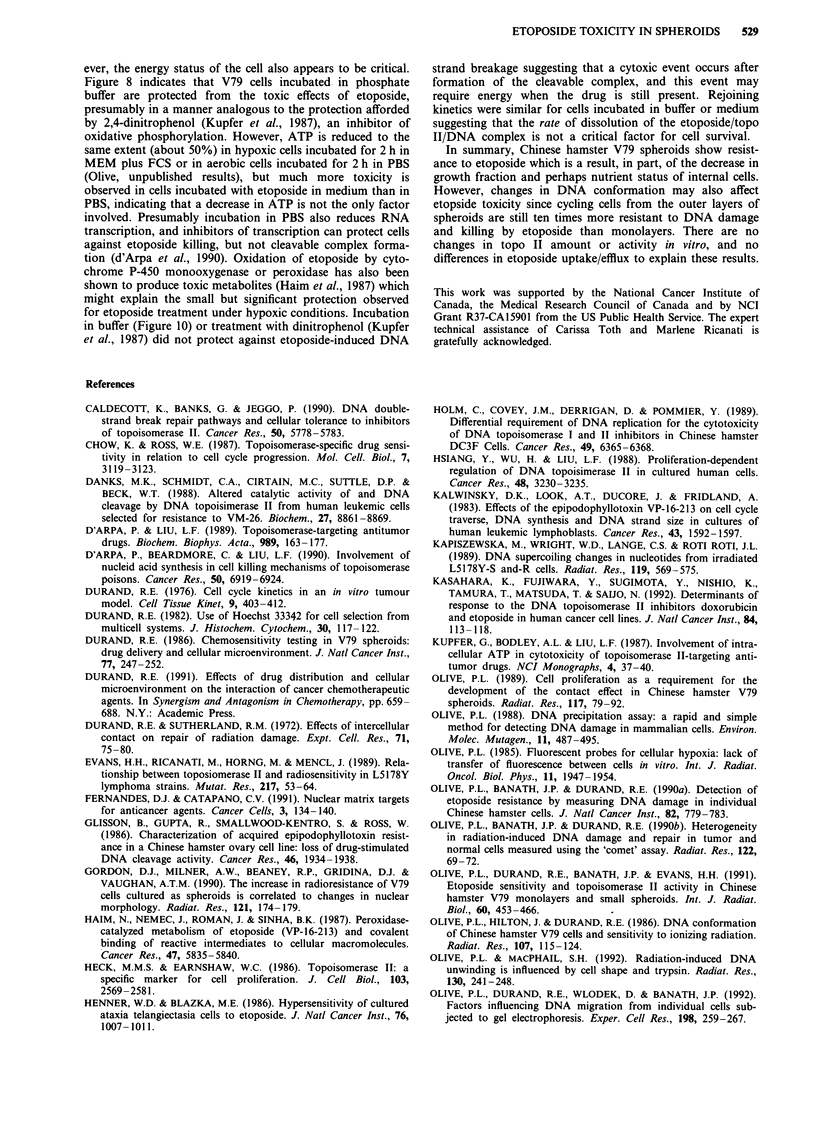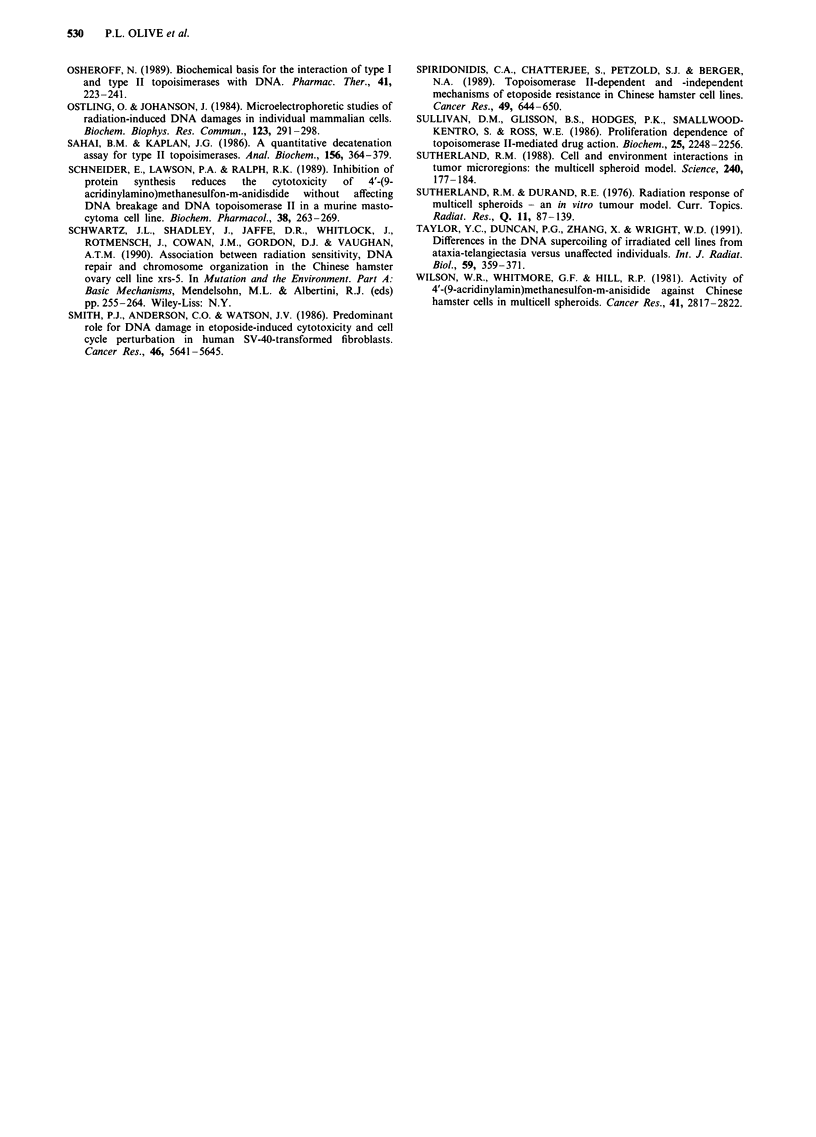# Cell killing and DNA damage by etoposide in Chinese hamster V79 monolayers and spheroids: influence of growth kinetics, growth environment and DNA packaging.

**DOI:** 10.1038/bjc.1993.97

**Published:** 1993-03

**Authors:** P. L. Olive, J. P. Banáth, H. H. Evans

**Affiliations:** British Columbia Cancer Research Center, Canada.

## Abstract

**Images:**


Br. J. Cancer (1993), 67, 522–530 © Macmillan Press Ltd., 1993

### Cell killing and DNA damage by etoposide in Chinese hamster V79 monolayers and spheroids: influence of growth kinetics, growth environment and DNA packaging

P.L. Olive[1], J.P. Banáth[1] & H.H. Evans[2]

[1]*British Columbia Cancer Research Center, 601 W. 10th Avenue, Vancouver, B.C. Canada V5Z 1L3;* [2]*Department of Radiology, Case Western Reserve University, Cleveland, Ohio 44106, USA.*

**Summary** Cells from V79 multicell spheroids must be exposed to approximately 50 times more etoposide than exponentially growing monolayers in order to produce the same amount of cell killing. A part of this difference in sensitivity is readily explained by the decrease in growth fraction of large spheroids, and by the protection afforded by nutrient deprivation which also reduces cellular ATP. However, cells composing the outer 10% of large (~600 µm diameter) V79 spheroids, although actively cycling, were still ten times more resistant to etoposide than exponentially growing monolayers, regardless of whether cells were exposed *in situ* in spheroids or dispersed by trypsin immediately prior to exposure to the drug. Four cell doublings (48 h) as monolayers were required before the outer cells of spheroids regained drug sensitivity equivalent to that of exponentially growing monolayers. No differences in uptake/efflux of $^3$H-etoposide or in levels of p-glycoprotein were observed between monolayers and the outer cells of spheroids. In addition, topoisomerase II protein measured by immunoblotting and topoisomerase II activity measured by decatenation of kinetoplast DNA were not reduced in the outer cells of spheroids compared to monolayers. DNA strand breakage measured in individual cells using the DNA precipitation and comet assays correlated well with cell killing with one exception: DNA damage was not affected when cells were incubated with etoposide in phosphate-buffered saline, although the etoposide concentration required to produce a given amount of cell killing was increased approximately 7-fold compared to cells incubated with the drug in complete medium. These results indicate that etoposide toxicity towards V79 spheroids is influenced not only by proliferative status of the cells but also by factors which may include DNA packaging and the growth environment of the cell prior to and during treatment.

Etoposide, a derivative of podophyllotoxin, is an effective antitumour agent which is selectively toxic to proliferating cells (Heck & Earnshaw, 1986; Sullivan *et al.*, 1986; Hsiang *et al.*, 1988). A major cause of toxicity is believed to be binding to etoposide to the nuclear enzyme, topoisomerase II (topo II), and trapping of this enzyme in a 'cleavable complex' with DNA; the resulting strand breaks can lead to cell killing (Osheroff, 1989; D'Arpa & Liu, 1989; Kalwinsky *et al.*, 1983). In a previous paper, we observed a 30% decrease in etoposide sensitivity to killing and DNA damage by etoposide when Chinese hamster V79 lung fibroblasts were grown for 24 h in suspension culture (Olive *et al.*, 1991). In suspension, V79 cells initially aggregate and then continue to grow as spheroids, and after 24 h, each spheroid is composed of 20–50 cells. After 10–14 days in suspension culture each spheroid is about 600 µm in diameter and contains about 100,000 cells (Sutherland & Durand, 1976). These large spheroids exhibited a 50-fold decrease in sensitivity to etoposide which we attributed at the time to a drop in growth fraction; as spheroids enlarge, the central cells stop proliferating and only the outer 2–3 cell layers continue to divide (Sutherland & Durand, 1976). Spheroid sensitivity to etoposide should therefore reflect damage to two populations, one retaining the sensitivity of the monolayer cells, and the other exhibiting resistance to DNA damage as well as to cell killing.

Previous studies with 4–7 day-old spheroids showed less etoposide-induced DNA damage than observed for monolayers, but the pattern of damage was inconsistent with the simple two component model described above (Olive *et al.*, 1991). The damaged population showed much less DNA breakage than expected on the basis of the monolayer response, and the resistance could not be explained completely by a decrease in growth fraction. We therefore examined etoposide-induced cell killing in cells recovered from different depths within intact and dissociated large spheroids. DNA damage was measured using the 'comet' assay, a method originally described by Ostling and Johanson (1984) and modified by us to quantify DNA single strand breaks (Olive *et al.*, 1990*a*,*b*). This method measures DNA strand breaks produced in *individual* cells, and is therefore the method of choice when studying heterogeneous systems such as multicell spheroids. Since the inner cells of spheroids also lack oxygen and nutrients (Sutherland & Durand, 1976), V79 cell sensitivity to etoposide was also examined in hypoxic and nutrient-deprived single cells. Western blotting and decatenation of kinetoplast DNA by proteins recovered from monolayers and spheroids were measured since resistance to etoposide has often been correlated with a change in topo II content or activity (Kasahara *et al.*, 1992; Danks *et al.*, 1988; Glisson *et al.*, 1986; Spiridonidis *et al.*, 1989).

### Materials and methods

### *Cell and spheroid culture*

Chinese hamster V79-171b lung fibroblasts were maintained in exponential growth by subcultivation twice weekly in Eagle's minimal essential medium (MEM) containing 10% fetal bovine serum (FBS). Spheroids were initiated by seeding $5 \times 10^4$ cells ml$^{-1}$ into Bellco spinner culture vessels containing MEM plus 5% FBS. Monolayers prepared for the same experiment were seeded at the same density on Falcon 10 mm tissue culture dishes in MEM plus 5% FBS. Larger spheroids were fed after 3 days and daily thereafter with medium supplemented with antibiotics.

### *Drug treatment and toxicity*

For experiments, monolayers and 1-day-old spheroids were trypsinised and resuspended in 10 ml complete medium in Petri dishes at a density of $5 \times 10^5$ cells/100 mm dish.

Correspondence: P.L. Olive.
Received 8 September 1992; and in revised form 4 November 1992.

Spheroids were exposed to etoposide in suspension either as intact spheroids or as dissociated spheroids, using the same cell density for exposure. Etoposide was prepared immediately before each experiment by adding the drug directly from the stock solution (purchased from Bristol Meyers, Canada Inc.) to complete medium, with vigorous shaking. Incubation was carried out at 37°C. Individual cells were plated in fresh medium for clonogenicity measurements, and approximately 1,000 colonies were counted per dose point in each experiment. For experiments which examined cell sensitivity as a function of time after dissociation of spheroids, cells were plated at different densities to ensure approximately $5 \times 10^5$ cells/100 mm dish at the time of drug exposure.

Selection of cells from various depths within spheroids was accomplished using two methods: Hoechst 33342 cell sorting as previously described (Durand, 1982), and sequential trypsinisation. Briefly, 12-day old V79 spheroids (approximately 600 μm in diameter) were incubated for 20 min with 1 μg ml$^{-1}$ Hoechst 33342, a fluorescent dye which penetrates poorly into multicell spheroids; the dye remains localised following disaggregation of the spheroid using trypsin. Cells can then be sorted on the basis of Hoechst 33342 concentration using a Becton-Dickinson FACS 440 dual laser cell sorter with the expectation that the brightly fluorescent cells are the outer cells of the spheroid, and the dimly fluorescent cells are those cells from the inner layers of the spheroid (Durand, 1982). Ten fractions, each representing 10% of the spheroid, were routinely sorted using sterile technique, and cells were plated from sorting tubes directly into tissue culture dishes for measurement of clonogenicity. Spheroids incubated with the drug after dissociation were first exposed to Hoechst 33342 for 20 min, followed by disaggregation and exposure for 1 h to etoposide. For some experiments, cells from different depths within spheroids were obtained by exposing spheroids for 5 min to 0.1% trypsin at 15°C while shaking in small culture dishes on an orbital platform. This reproducibly released 8–14% of the cells from the outer layer(s) of the spheroids. The remaining cells were also examined for topo II activity. For hypoxic incubations with etoposide, V79 cells ($2 \times 10^5$ cells ml$^{-1}$) were incubated in glass spinner culture vessels under constant gassing with either oxygen-free nitrogen for treatments in phosphate-buffer saline (PBS), or certified nitrogen plus 5% $CO_2$ for incubations in medium. Solutions were equilibrated with the gas for 1 h prior to addition of the cells; gassing in combination with oxygen consumption by the cells was sufficient to reduce the oxygen content below 100 p.p.m. (Olive, 1985). Cell survival experiments comparing aerobic and hypoxic incubations were repeated 4–7 times and the mean ± standard error determined.

*DNA damage measured using the alkaline comet assay*

For the alkaline comet assay, single cells were centrifuged immediately after the 1 h exposure to etoposide and resuspended in ice-cold phosphate-buffered saline (PBS) at a concentration of $2-4 \times 10^4$ cells ml$^{-1}$. 0.5 ml cell suspension ($10^4$ cells) was placed in a 5 ml disposable tube and 1.5 ml 1% low gelling temperature agarose (Sigma type VII prepared in distilled water and held at 40°C), was added to the tube. The contents were quickly pipetted onto a half-frosted microscope slide and allowed to gel for about 1 min on a cold surface. Slides were carefully submersed in an alkaline lysis solution containing 1 M NaCl and 0.03 M NaOH for 1 h followed by a 1 h wash in 0.03 M NaOH, 2 mM EDTA before electrophoresis in a fresh solution of 0.03 M NaOH, 2 mM EDTA at 0.5 volt cm$^{-1}$ for 25 min. Slides were rinsed and stained for 10 min in 2.5 μg ml$^{-1}$ propidium iodide.

Individual cells or 'comets' were viewed using a Zeiss epifluorescence microscope attached to an intensified solid state CCD camera and image analysis system. For viewing propidium iodide fluorescence, slides were illuminated with green light from a 100 watt mercury source using a 580 nm reflector and 590 nm barrier filter. Individual comets were viewed using a 25 × objective and images were analysed using a fluorescence image processing system previously described (Olive *et al.*, 1990*b*). The 'tail moment', defined as the product of the percentage of DNA in the tail multiplied by the tail length, and 'DNA content', defined as the total fluorescence associated with an image, were the most informative features (Olive *et al.*, 1990*b*).

*DNA precipitation assay*

The alkaline DNA precipitation assay described previously (Olive, 1988) was used to detect DNA single-strand breaks produced by etoposide. This method measures the fraction of DNA which precipitates when sodium dodecyl sulphate (SDS) at pH 12.3 lyses the cells and potassium chloride is added to cause precipitation of the detergent, cellular protein and large molecular weight DNA. In this method, there is no shearing of the DNA, and SDS at pH 12.3 effectively removes proteins (like topo II) bound to DNA.

*Measurement of etoposide uptake/efflux and C219 binding*

Uptake and efflux of etoposide was measured using $^3$H-etoposide, specific activity 14.8 GBq/mmol, obtained from Moravek Biochemicals, Brea, CA. $4 \times 10^5$ cells from exponentially growing monolayers or from the outer 8–12% of the cells of V79 spheroids were allowed to attach for 2 h in 24-well dishes. Etoposide (37 kBq in 1 ml) was added to the dishes, and after 60 min, wells were rinsed twice and fresh medium was added at 37°C. At specified times later, medium was aspirated and SDS was added to the wells. Solutions were transferred to scintillation vials along with the rinse solution for radioactivity measurement in a LKB liquid scintillation counter. Assays were performed in duplicate and repeated 2–3 times.

P-glycoprotein was measured in monolayers and the outer cells of spheroids using an antibody (C219) purchased from Centocor (Malvern, PA). Methods used for flow cytometry evaluation of antibody binding were those recommended by the supplier and included incubation of a fixed cell sample with a 'negative' antibody to obtain background fluorescence.

*Determination of topoisomerase II activity*

The activity of topoisomerase II in homogenates of V79 cells was measured using a modification (Olive *et al.*, 1991) of the trypanosome kinetoplast DNA decatenation reaction described by Sahai and Kaplan (1986). In some experiments, various amounts of etoposide were added to the homogenates (0.75 mg kg$^{-1}$ protein and 0.25 μg ml$^{-1}$ $^3$H-kinetoplast DNA) before incubation. For these experiments only, etoposide (obtained as a powder from Bristol Meyers) was dissolved in DMSO and equal amounts of DMSO were added to all samples, including the controls. Topoisomerase II activity was measured by the percentage of kinetoplast DNA decatenated after a 20 min incubation at 30°C. Cell homogenates and $^3$H-kinetoplast DNA were prepared as described previously (Olive *et al.*, 1991).

*Topisomerase II measured by immunoblotting*

Immunoblotting was performed on nuclear proteins from monolayers and 1-day-old spheroids. The same number of nuclei obtained by sequential trypsinisation from Chinese hamster V79 monolayers, and the outer ~10%, middle ~20% and inner ~70% of cells of spheroids were lysed in sample loading buffer. SDS-polyacrilamide gel electrophoresis and transfer of proteins to nitrocellulose was performed as previously described (Olive & MacPhail, 1992). High-molecular weight rainbow markers from Bio-rad were also transferred to nitrocellulose. Blots were blocked by a 2 h incubation with 5% skim milk in PBS. Topoisomerase II was detected by incubation with rabbit antibodies against human recombinant topoisomerase II (c-terminal), kindly supplied by Dr Leroy Liu. A 1:500 dilution of the antibodies was prepared in PBS containing 3% BSA. After incubation overnight at room temperature, blots were washed in 3% bovine serum albumin in PBS followed by incubation for 1 h with peroxide conjugated goat anti-rabbit IgG. Colour formation by horseradish peroxidase was induced by soaking the blots in a solution of 600 μg ml$^{-1}$ of 3-3'diaminobenzidine with 0.3% hydrogen peroxide in 50 mM Tris, pH 7.6. The reaction was stopped by rinsing with PBS. Experiments were repeated using three separate populations of spheroids and monolayers, and a representative blot is shown.

### Results

A large difference was observed in the etoposide sensitivity of the internal *vs* the external cells of Chinese hamster V79 spheroids (Figure 1). This was expected since the internal cells are non-cycling (Durand, 1976) and should therefore not respond to etoposide (Heck & Earnshaw, 1986; Sullivan *et al.*, 1986; Hsiang, 1988). However, the external cells of 600 μm diameter Chinese hamster V79 spheroids were approximately ten times more resistant to etoposide than exponentially growing monolayers, and the sensitivity of these cells to etoposide was similar whether spheroids were incubated with the drug intact or dissociated prior to treatment (Figure 1). The resistance of the external cells of V79 spheroids is difficult to explain since almost all of these cells are cycling, well-nourished, and have direct access to the drug. When V79 cells are placed in suspension culture, they aggregate and begin to grow as multicell spheroids. Resistance to etoposide develops rapidly over the first 3 days in culture, and then remains fairly constant (Figure 2a). The mitotic index and labelling index drop rapidly between 2–6 days in culture, accounting for some of this increased resistance, although the cell cycle time, measured using $^{3}$H-thymidine labelling does not change significantly (Sutherland & Durand, 1976; Durand, 1976). Recent flow cytometry measurements of transit times of cells incorporating bromodeoxyuridine confirms that S phase durations for the proliferating cells of spheroids (~10 h) are similar to those observed for exponentially growing monolayers (R. Durand, personal communication). However, as shown in Figure 1 and 2b, the external cycling cells from larger spheroids are also more resistant to killing by etoposide, so the change in growth kinetics is not the explanation for the resistance of these outer cells. In fact, when the outer cells were separatec from the spheroids using sequential trypsinisation anc examined for their sensitivity to etoposide as a function ol time after return to monolayer growth, sensitivity did noi change significantly over the first 8 h in culture, but subsequently increased over the next 40 h (Figure 2b). The time tc

**Figure 1** Toxicity of etoposide to V79-171b monolayers and large spheroids. Spheroids (approximately 600 μm diameter) were incubated with etoposide either intact (●,▼) or dissociated prior to treatment (Δ,□). Monolayers (○) were incubated with the drug while attached to petri dishes. In all cases, cell density was kept constant at 5 × 10$^{4}$ cells ml$^{-1}$ during the 1 h treatment. The inner 10% (●,▽) and outer 10% (▼,□) of cells of spheroids were sorted on the basis of the Hoechst 33342 fluorescence gradient (Durand, 1982). Lines are drawn using the linear-quadratic formula. Symbols with error bars show the means and standard error for three experiments.

**Figure 2** Toxicity of etoposide as a function of spheroid age. **a**, Etoposide toxicity to intact spheroids (2 h exposure) was measured as a function of time of growth as spheroids. Panel **b**, the outer 8–10% of cells were recovered from 11-day-old spheroids using sequential trypsinisation and replated as monolayers. Etoposide toxicity (1 h exposure) is measured as a function of time after return to monolayer growth. ●, 2 μg ml$^{-1}$; ▽, 5 μg ml$^{-1}$; ▼, 10 μg ml$^{-1}$ etoposide.

return to monolayer sensitivity was equivalent to about four cell doublings. During this entire period, cells grew exponentially, with a doubling time of 12 h, identical to that for exponentially-growing monolayers.

To understand why the external cells of V79 cells are more resistant to etoposide than monolayers, DNA strand breakage following exposure of monolayers and spheroids was measured in individual cells using a sensitive microelectrophoresis method called the 'comet assay'. We have previously shown that the number of DNA single-strand breaks is related to the extent of DNA migration (Olive *et al.*, 1990*b*), and etoposide is effective in producing strand breaks measured using this method (Olice *et al.*, 1990*a*). As expected, the non-dividing internal cells of spheroids were resistant to etoposide-induced DNA damage (Figure 3). However, the external cells of V79 spheroids were also about six times more resistant than exponentially growing monolayers. This results is in reasonably good agreement with the toxicity results shown in Figure 1 considering that our endpoint for DNA damage, 'tail moment', emphasises the response of the damaged cells, not the surviving cells.

The resistance of the outer cells of spheroids to DNA damage by etoposide could result if two populations were present in the external cells of spheroids: one population equally as sensitive as the monolayers, and the other population responding like non-cycling cells. By examining the heterogeneity of tail moment measured for individual cells, this question can be resolved. In spite of the large heterogeneity, it is clear from histograms shown in Figure 4 that the entire population of outer cells is considerably more resistant to DNA damage than monolayers. A small fraction of the outer cells do not respond to etoposide (these cells are the survivors), but the distribution of comet tail moments for the external 10% of cells of V79 spheroids exposed to 2 $\mu$g ml$^{-1}$ etoposide did not overlap significantly the distribution for exponentially growing monolayers exposed to this concentration. More heterogeneity in DNA damage was observed after etoposide treatment (Figure 4) than after X-ray treatment (Olive *et al.*, 1992). This does not appear to be a result of inherent differences in DNA damage through the cell cycle since tail moment was independent of DNA content measured simultaneously for each comet (Figure 5). Permeabilisation using saponin did not reduce heterogeneity or the 10-fold difference between the sensitivity of monolayers and the outer cells of spheroids to etoposide. Therefore, both clonogenicity and DNA damage assays indicate a substantial difference in the sensitivity of monolayers and the outer cells of spheroids which cannot be explained by a decrease in growth fraction.

**Figure 4** Heterogeneity in DNA damage detected using the alkaline comet assay. Monolayers and 600 $\mu$m diameter spheroids were incubated for 1 h with 2 $\mu$g ml$^{-1}$ etoposide. The outer and inner cells of the spheroid were then separated by cell sorting. Results show the distribution of tail moments for 100 individual cells.

**Figure 3** DNA damage to V79 monolayers and spheroids measured using the alkaline comet assay. Intact spheroids were incubated with etoposide followed by exposure to Hoechst 33342. Spheroids were then trypsinised and cells were sorted on the basis of the Hoechst 33342 diffusion gradient into populations representing the outer 10% or the inner 10% of the spheroids. The tail moment is a measure of the number of single strand breaks present in an individual cell. The mean and standard error for three independent experiments (40–100 individual comets/point/experiment) are shown.

The ability of the comet assay to predict cell sensitivity to etoposide is shown in Figure 6. Here the tail moment was measured in populations of monolayers and spheroids exposed to etoposide, and the fraction of comets with tail moments less than 2.0 were defined to be viable cells since 95% of the untreated comets had tail moments <2.0. The linear correlation between DNA damage and survival shown in Figure 6b (using data from Figure 6a and Figure 1) indicates that DNA damage in both monolayers and spheroids is predictive for cell survival. The mechanism of cell killing by etoposide seems to be the same for monolayers and spheroids.

No significant differences in efflux of $^{3}$H-etoposide were detected between monolayers and the external cells of spheroids removed by a short exposure to trypsin (Figure 7). The drug diffused rapidly into and out of cells with very little of the radioactivity being retained; results were highly dependent on speed of rinsing. Similarly, there was no difference in p-glycoprotein, as determined by binding of antibodies against gp 170 by monolayers and the external cells of spheroids; in both cases, mean cellular fluorescence after exposure to C219 antibody was 40% greater than after exposure to a non-specific antibody (data not shown).

Similar amounts of topo II protein, measured by immunoblotting, were present in monolayers, the outer ~10% and middle ~20% of cells of spheroids (Figure 8). However protein was significantly reduced in remaining ~70% of the cells of the spheroid. In all cases, the 170 kD form of topo II predominated, and the pattern of antibody binding was the same for all samples.

**Figure 5** Bivariate plot comparing DNA content and tail moment measured for monolayers exposed to etoposide. Exponentially growing V79 monolayers were exposed to 2 $\mu g\ ml^{-1}$ etoposide for 1 h followed by examination of DNA damage using the comet assay. DNA content (total fluorescence) is compared to tail moment measured for the same comet. Data for 200 individual cells are shown.

Topo II activity, as measured by decatenation of kinetoplast DNA, was not significantly reduced in the outer 10%–15% of cells of spheroids compared to monolayers (Figure 9a). However, the remaining ~90% of spheroid cells (minus the outer cells) indicated the anticipated reduction in topo II activity; this population would be expected to contain about 30–40% cycling cells since the growth fraction of V79 spheroids of this size is about 40–50% (Durand, 1976). Also, as shown in Figure 8, the middle layers of spheroids do contain significant amounts of topo II. Etoposide added to the reaction mixture reduced the amount of decatenation

**Figure 7** Efflux of $^3$H-etoposide from V79 monolayers (O) and the outer 8–12% of cells from ~600 $\mu m$ diameter spheroids (x) prepared by sequential trypsinisation. The mean and standard error for three experiments is shown.

**Figure 6** Prediction of cell survival using the comet assay. **a**, Monolayers (x), inner (△,▲) and outer (O,●) cells of spheroids were analysed for DNA damage following a 1 h exposure of intact (●,▲) or disaggregated (O,△) spheroids. The mean ± standard error are shown for three determinations. The fraction of comets with tail moments less than 2.0 is plotted as a function of etoposide concentration. **b**, Results in Figure 1 are compared to results in panel **a**.

**Figure 8** Topo II protein measured by immunoblotting. Topo II proteins (bands at ~170 kD and ~180 kD) were measured in nuclei prepared by sequential trypsinisation from the inner ~70% of spheroids (lane **a**), the outer ~10% of spheroids **b**, monolayers **c**, or the middle ~20% of spheroids **d**.

(Figure 9b). However this reduction, expressed as a percent of the decatenation observed in untreated samples, was similar for monolayers, outer cells and remaining cells of spheroids.

Less killing was produced in the internal than external cells of intact spheroids, but sensitivity of the inner cells was increased when spheroids were disaggregated prior to etoposide treatment (Figure 1). The internal cells in intact spheroids have less access to oxygen and nutrients (Sutherland & Durand, 1976) and may be protected from damage because of a reduced energy status; once the spheroid is disaggregated, protection may be lost immediately. This explanation is supported by results shown in Figure 10; about seven times less etoposide was required to produce the same surviving fraction for cells incubated with the drug dissolved in medium equilibrated with air compared to cells incubated with the drug dissolved in buffer equilibrated with nitrogen. This difference was primarily due to incubation of cells in PBS since hypoxic cells were only about 30% more resistant to etoposide than well-oxygenated cells when incubated in buffer or medium; this difference was significant at the 5% level using a paired *t*-test. No significant difference in toxicity was observed for V79 cells incubated with etoposide in medium equilibrated at pH 6.5 to 7.4 (data not shown).

Interestingly, the decreased cell killing in buffer was not accompanied by a decrease in DNA damage by etoposide. In fact, aerobic cells incubated with etoposide in PBS showed a small relative increase in DNA single-strand breakage measured using the DNA precipitation assay (Figure 9b). Rejoining of DNA breaks after a 2 h exposure to etoposide was identical for cells exposed to etoposide in MEM or PBS (Figure 9c). A similar dissociation between cell killing and DNA damage was not, however, observed with the inner cells of spheroids which are also presumably nutrient deficient; both cell killing and DNA damage (Figure 6a) were increased when spheroids were disaggregated prior to exposure to etoposide.

### Discussion

Multicell spheroids are often used to model features of the tumour cell microenvironment in order to examine factors which might influence the response of tumour cells to therapeutic agents (Sutherland, 1988; Durand, 1991; Durand & Sutherland, 1972). Close intercellular contact, cell cycle kinetics, drug accessibility and gradients of oxygen and nutrients may also act and interact to influence cell killing. It is clear that effects of chemotherapy agents on single cells may not predict for the response of cells in spheroids (Durand, 1986), and results shown here with etoposide support this conclusion.

The basis for this large difference between the sensitivity of monolayers and outer cells of V79 spheroids to etoposide is not yet known. The observation that, for approximately the same cell killing or DNA damage, the external cells of V79 spheroids must be exposed to ten times more etoposide than exponentially growing monolayers cannot be explained by differences in growth fraction or nutrient status. Even when growth fraction is discounted, there are likely to be many possible mechanisms of resistance to topoisomerase II poisons, but none of the proposed mechanisms can explain results with V79 spheroids. V79 spheroids do not overexpress p-glycoprotein, nor do we see differences in rate of uptake/efflux of etoposide. Etoposide was equally effective in decatenating trypanosome DNA when monolayer cell topo II was used as the enzyme source compared to topo II

**Figure 9** **a**, Topoisomerase II activity in homogenates of V79 cells. Homogenates of V79 monolayers (▼) and of the cells composing the outer 10–12% (●) or the remaining cells of 600 μ diameter spheroids (∇) obtained by sequential trypsinisation were examined for ability to decatenate 0.25 μg of 3H-kDNA as described in the Methods. **b**, Etoposide inhibition of topoisomerase II activity in homogenates of V79 cells. Indicated amounts of etoposide were added to the reaction mixture containing homogenates of V79 cells and $^3$H-kinetoplast DNA. Topo II activity was measured by the percentage of kinetoplast DNA decatenated after a 20 min incubation at 30°C. The activity of the homogenates treated with etoposide is plotted as the percentage of the activity of the untreated homogenates.

**Figure 10** Effect of culture conditions on etoposide toxicity and DNA damage. **a**, Toxicity of Etoposide to V79 monolayers incubated for 2 h. In all panels, the mean and standard error for three or more determinations is given. **b**, DNA single-strand breaks measured using the DNA precipitation assay. The mean and standard error for three determinations is given (damage producing 50% DNA precipitated is equivalent to 8 Gy). **c**, Repair of DNA damage following removal of etoposide and return of cells to complete medium. The mean and standard error for three determinations are shown. PBS, air (Δ); PBS, nitrogen (x); MEM, air (O); MEM, nitrogen (●).

recovered from the outer cells of spheroids (Figure 8b); this result suggests that topo II recovered from both cell types interacts in the same way with etoposide. We are therefore left with the possibility that the complex formed between etoposide, topo II and DNA is different in monolayers compared to the outer cells of spheroids.

Toxicity of another topoisomerase II inhibitor, m-AMSA, was examined several years ago in Chinese hamster V79 spheroids; results by Wilson et al. (1981) indicated that m-AMSA was significantly less toxic to the external cells of V79 spheroids than expected on the basis of the monolayer response. This result is important since it confirms our data with another inhibitor of topoisomerase II, and moreover, suggests that DNA intercalation, which is a property of m-AMSA but not etoposide, is not involved in this effect.

As we have discussed previously, there is an important similarity between the enhanced resistance of cells in V79 spheroids to killing by etoposide and the 'contact effect' described for ionising radiation-induced damage (Olive et al., 1991). We and others have suggested that differences in the sensitivity of monolayers and spheroids to ionising radiation is dependent upon the conformation or 'packaging' of chromatin (Olive et al., 1986; Olive, 1989; Gordon et al., 1990). If, as previous results suggests, DNA conformation and association with the nuclear matrix is altered in V79 spheroids, then this change could influence the response of cells to etoposide, a drug whose action requires the formation of a complex between itself, DNA and topoisomerase II. This hypothesis is supported by results from numerous studies which indicate a correlation between sensitivity to ionising radiation and sensitivity to topoisomerase II inhibitors for a variety of repair-deficient cell lines (Smith et al., 1986; Caldecott et al., 1990; Henner & Blazka, 1986; Evans et al., 1989). For all of these cell lines, changes in DNA conformation have been implicated in their increased sensitivity to ionising radiation (Schwartz et al., 1990; Taylor et al., 1991; Kapiszewska et al., 1989). It therefore seems likely that DNA conformational differences can influence sensitivity to etoposide. Recent studies indicating that the nature of attachment/association of topo II to the nuclea matrix can be cell line dependent and can influence susceptibility to topo II poisons provides further support for this argument (Fernandes & Catapano, 1991).

The relation between cell killing and DNA damage by etoposide was similar for monolayers and spheroids (Figure 6), a result that could be interpreted to mean that the mechanism of cell killing by etoposide is similar for these two populations. However, this correlation results because a small fraction of the outer 10% of cells of the V79 spheroids do not show significant DNA damage probably because they are non-cycling. Additional evidence for the presence of non-cycling cells in the external cells layers comes from data in Figure 1 where the curve for external cells from intact spheroids appears to flatten at higher etoposide concentrations. Extrapolation of this curve back to the ordinate suggests that a non-cycling fraction as high as 10% could be present in the outer cell layer. These non-responding cells are the ones that survive exposure to etoposide. If average DNA damage is compared to cell survival, the correlation is not as good, and can be compared to results of Spiridonidas and coworkers (1989) who also examined the relation between survival and DNA damage for etoposide-resistant V79 cell lines. They observed more cell killing than expected on the basis of average number of DNA strand breaks/cell. However, survival reflects the fraction of the population with the least amount of damage, so that a method that examines DNA damage in individual cells is an important advantage in making these kinds of comparisons. We expected to detect, in addition to these resistant cells, at least some external cells of the spheroids with DNA damage equivalent to the level sustained by monolayers. As shown in Figure 4, there was no evidence that a significant fraction of the outermost spheroid cells were as sensitive to 2 $\mu g\, ml^{-1}$ etoposide as monolayers. In other words, while a proportion (we estimate to be about 4–10% from Figure 5 and similar data) of the external cell layer may not be cycling and will therefore survive etoposide treatment, the remaining cells are considerably more resistant than expected to DNA damage by etoposide.

Several authors have noted a discrepancy between etoposide-induced cleavable complex formation and cell killing which indicates that factors in addition to trapping of complexes are involved in cell inactivation (Chow & Ross, 1987; Schneider et al., 1989; Holm et al., 1989). As discussed above, DNA 'packaging' may be one of these factors, however, the energy status of the cell also appears to be critical. Figure 8 indicates that V79 cells incubated in phosphate buffer are protected from the toxic effects of etoposide, presumably in a manner analogous to the protection afforded by 2,4-dinitrophenol (Kupfer *et al*., 1987), an inhibitor of oxidative phosphorylation. However, ATP is reduced to the same extent (about 50%) in hypoxic cells incubated for 2 h in MEM plus FCS or in aerobic cells incubated for 2 h in PBS (Olive, unpublished results), but much more toxicity is observed in cells incubated with etoposide in medium than in PBS, indicating that a decrease in ATP is not the only factor involved. Presumably incubation in PBS also reduces RNA transcription, and inhibitors of transcription can protect cells against etoposide killing, but not cleavable complex formation (d'Arpa *et al*., 1990). Oxidation of etoposide by cytochrome P-450 monooxygenase or peroxidase has also been shown to produce toxic metabolites (Haim *et al*., 1987) which might explain the small but significant protection observed for etoposide treatment under hypoxic conditions. Incubation in buffer (Figure 10) or treatment with dinitrophenol (Kupfer *et al*., 1987) did not protect against etoposide-induced DNA strand breakage suggesting that a cytoxic event occurs after formation of the cleavable complex, and this event may require energy when the drug is still present. Rejoining kinetics were similar for cells incubated in buffer or medium suggesting that the *rate* of dissolution of the etoposide/topo II/DNA complex is not a critical factor for cell survival.

In summary, Chinese hamster V79 spheroids show resistance to etoposide which is a result, in part, of the decrease in growth fraction and perhaps nutrient status of internal cells. However, changes in DNA conformation may also affect etopside toxicity since cycling cells from the outer layers of spheroids are still ten times more resistant to DNA damage and killing by etoposide than monolayers. There are no changes in topo II amount or activity *in vitro*, and no differences in etoposide uptake/efflux to explain these results.

This work was supported by the National Cancer Institute of Canada, the Medical Research Council of Canada and by NCI Grant R37-CA15901 from the US Public Health Service. The expert technical assistance of Carissa Toth and Marlene Ricanati is gratefully acknowledged.

### References

CALDECOTT, K., BANKS, G. & JEGGO, P. (1990). DNA double-strand break repair pathways and cellular tolerance to inhibitors of topoisomerase II. *Cancer Res*., **50**, 5778–5783.

CHOW, K. & ROSS, W.E. (1987). Topoisomerase-specific drug sensitivity in relation to cell cycle progression. *Mol. Cell. Biol*., **7**, 3119–3123.

DANKS, M.K., SCHMIDT, C.A., CIRTAIN, M.C., SUTTLE, D.P. & BECK, W.T. (1988). Altered catalytic activity of and DNA cleavage by DNA topoisimerase II from human leukemic cells selected for resistance to VM-26. *Biochem*., **27**, 8861–8869.

D'ARPA, P. & LIU, L.F. (1989). Topoisomerase-targeting antitumor drugs. *Biochem. Biophys. Acta*., **989**, 163–177.

D'ARPA, P., BEARDMORE, C. & LIU, L.F. (1990). Involvement of nucleid acid synthesis in cell killing mechanisms of topoisomerase poisons. *Cancer Res*., **50**, 6919–6924.

DURAND, R.E. (1976). Cell cycle kinetics in an *in vitro* tumour model. *Cell Tissue Kinet*, **9**, 403–412.

DURAND, R.E. (1982). Use of Hoechst 33342 for cell selection from multicell systems. *J. Histochem. Cytochem*., **30**, 117–122.

DURAND, R.E. (1986). Chemosensitivity testing in V79 spheroids: drug delivery and cellular microenvironment. *J. Natl Cancer Inst*., **77**, 247–252.

DURAND, R.E. (1991). Effects of drug distribution and cellular microenvironment on the interaction of cancer chemotherapeutic agents. In *Synergism and Antagonism in Chemotherapy*, pp. 659–688. N.Y.: Academic Press.

DURAND, R.E. & SUTHERLAND, R.M. (1972). Effects of intercellular contact on repair of radiation damage. *Expt. Cell. Res*., **71**, 75–80.

EVANS, H.H., RICANATI, M., HORNG, M. & MENCL, J. (1989). Relationship between toposiomerase II and radiosensitivity in L5178Y lymphoma strains. *Mutat. Res*., **217**, 53–64.

FERNANDES, D.J. & CATAPANO, C.V. (1991). Nuclear matrix targets for anticancer agents. *Cancer Cells*, **3**, 134–140.

GLISSON, B., GUPTA, R., SMALLWOOD-KENTRO, S. & ROSS, W. (1986). Characterization of acquired epipodophyllotoxin resistance in a Chinese hamster ovary cell line: loss of drug-stimulated DNA cleavage activity. *Cancer Res*., **46**, 1934–1938.

GORDON, D.J., MILNER, A.W., BEANEY, R.P., GRIDINA, D.J. & VAUGHAN, A.T.M. (1990). The increase in radioresistance of V79 cells cultured as spheroids is correlated to changes in nuclear morphology. *Radiat. Res*., **121**, 174–179.

HAIM, N., NEMEC, J., ROMAN, J. & SINHA, B.K. (1987). Peroxidase-catalyzed metabolism of etoposide (VP-16-213) and covalent binding of reactive intermediates to cellular macromolecules. *Cancer Res*., **47**, 5835–5840.

HECK, M.M.S. & EARNSHAW, W.C. (1986). Topoisomerase II: a specific marker for cell proliferation. *J. Cell Biol*., **103**, 2569–2581.

HENNER, W.D. & BLAZKA, M.E. (1986). Hypersensitivity of cultured ataxia telangiectasia cells to etoposide. *J. Natl Cancer Inst*., **76**, 1007–1011.

HOLM, C., COVEY, J.M., DERRIGAN, D. & POMMIER, Y. (1989). Differential requirement of DNA replication for the cytotoxicity of DNA topoisomerase I and II inhibitors in Chinese hamster DC3F Cells. *Cancer Res*., **49**, 6365–6368.

HSIANG, Y., WU, H. & LIU, L.F. (1988). Proliferation-dependent regulation of DNA topoisimerase II in cultured human cells. *Cancer Res*., **48**, 3230–3235.

KALWINSKY, D.K., LOOK, A.T., DUCORE, J. & FRIDLAND, A. (1983). Effects of the epipodophyllotoxin VP-16-213 on cell cycle traverse, DNA synthesis and DNA strand size in cultures of human leukemic lymphoblasts. *Cancer Res*., **43**, 1592–1597.

KAPISZEWSKA, M., WRIGHT, W.D., LANGE, C.S. & ROTI ROTI, J.L. (1989). DNA supercoiling changes in nucleotides from irradiated L5178Y-S and-R cells. *Radiat. Res*., **119**, 569–575.

KASAHARA, K., FUJIWARA, Y., SUGIMOTA, Y., NISHIO, K., TAMURA, T., MATSUDA, T. & SAIJO, N. (1992). Determinants of response to the DNA topoisomerase II inhibitors doxorubicin and etoposide in human cancer cell lines. *J. Natl Cancer Inst*., **84**, 113–118.

KUPFER, G., BODLEY, A.L. & LIU, L.F. (1987). Involvement of intracellular ATP in cytotoxicity of topoisomerase II-targeting antitumor drugs. *NCI Monographs*, **4**, 37–40.

OLIVE, P.L. (1989). Cell proliferation as a requirement for the development of the contact effect in Chinese hamster V79 spheroids. *Radiat. Res*., **117**, 79–92.

OLIVE, P.L. (1988). DNA precipitation assay: a rapid and simple method for detecting DNA damage in mammalian cells. *Environ. Molec. Mutagen*., **11**, 487–495.

OLIVE, P.L. (1985). Fluorescent probes for cellular hypoxia: lack of transfer of fluorescence between cells *in vitro*. *Int. J. Radiat. Oncol. Biol. Phys*., **11**, 1947–1954.

OLIVE, P.L., BANATH, J.P. & DURAND, R.E. (1990*a*). Detection of etoposide resistance by measuring DNA damage in individual Chinese hamster cells. *J. Natl Cancer Inst*., **82**, 779–783.

OLIVE, P.L., BANATH, J.P. & DURAND, R.E. (1990*b*). Heterogeneity in radiation-induced DNA damage and repair in tumor and normal cells measured using the 'comet' assay. *Radiat. Res*., **122**, 69–72.

OLIVE, P.L., DURAND, R.E., BANATH, J.P. & EVANS, H.H. (1991). Etoposide sensitivity and topoisomerase II activity in Chinese hamster V79 monolayers and small spheroids. *Int. J. Radiat. Biol*., **60**, 453–466.

OLIVE, P.L., HILTON, J. & DURAND, R.E. (1986). DNA conformation of Chinese hamster V79 cells and sensitivity to ionizing radiation. *Radiat. Res*., **107**, 115–124.

OLIVE, P.L. & MACPHAIL, S.H. (1992). Radiation-induced DNA unwinding is influenced by cell shape and trypsin. *Radiat. Res*., **130**, 241–248.

OLIVE, P.L., DURAND, R.E., WLODEK, D. & BANATH, J.P. (1992). Factors influencing DNA migration from individual cells subjected to gel electrophoresis. *Exper. Cell Res*., **198**, 259–267.

OSHEROFF, N. (1989). Biochemical basis for the interaction of type I and type II topoisimerases with DNA. *Pharmac. Ther.*, **41,** 223–241.

OSTLING, O. & JOHANSON, J. (1984). Microelectrophoretic studies of radiation-induced DNA damages in individual mammalian cells. *Biochem. Biophys. Res. Commun.*, **123,** 291–298.

SAHAI, B.M. & KAPLAN, J.G. (1986). A quantitative decatenation assay for type II topoisimerases. *Anal. Biochem.*, **156,** 364–379.

SCHNEIDER, E., LAWSON, P.A. & RALPH, R.K. (1989). Inhibition of protein synthesis reduces the cytotoxicity of 4'-(9-acridinylamino)methanesulfon-m-anidisdide without affecting DNA breakage and DNA topoisomerase II in a murine mastocytoma cell line. *Biochem. Pharmacol.*, **38,** 263–269.

SCHWARTZ, J.L., SHADLEY, J., JAFFE, D.R., WHITLOCK, J., ROTMENSCH, J., COWAN, J.M., GORDON, D.J. & VAUGHAN, A.T.M. (1990). Association between radiation sensitivity, DNA repair and chromosome organization in the Chinese hamster ovary cell line xrs-5. In *Mutation and the Environment. Part A: Basic Mechanisms*, Mendelsohn, M.L. & Albertini, R.J. (eds) pp. 255–264. Wiley-Liss: N.Y.

SMITH, P.J., ANDERSON, C.O. & WATSON, J.V. (1986). Predominant role for DNA damage in etoposide-induced cytotoxicity and cell cycle perturbation in human SV-40-transformed fibroblasts. *Cancer Res.*, **46,** 5641–5645.

SPIRIDONIDIS, C.A., CHATTERJEE, S., PETZOLD, S.J. & BERGER, N.A. (1989). Topoisomerase II-dependent and -independent mechanisms of etoposide resistance in Chinese hamster cell lines. *Cancer Res.*, **49,** 644–650.

SULLIVAN, D.M., GLISSON, B.S., HODGES, P.K., SMALLWOOD-KENTRO, S. & ROSS, W.E. (1986). Proliferation dependence of topoisomerase II-mediated drug action. *Biochem.*, **25,** 2248–2256.

SUTHERLAND, R.M. (1988). Cell and environment interactions in tumor microregions: the multicell spheroid model. *Science*, **240,** 177–184.

SUTHERLAND, R.M. & DURAND, R.E. (1976). Radiation response of multicell spheroids – an *in vitro* tumour model. Curr. Topics. *Radiat. Res.*, **Q. 11,** 87–139.

TAYLOR, Y.C., DUNCAN, P.G., ZHANG, X. & WRIGHT, W.D. (1991). Differences in the DNA supercoiling of irradiated cell lines from ataxia-telangiectasia versus unaffected individuals. *Int. J. Radiat. Biol.*, **59,** 359–371.

WILSON, W.R., WHITMORE, G.F. & HILL, R.P. (1981). Activity of 4'-(9-acridinylamin)methanesulfon-m-anisidide against Chinese hamster cells in multicell spheroids. *Cancer Res.*, **41,** 2817–2822.